# Environmental Context Indicator for Evaluating Quality of GNSS Observation Environment Using Android Smartphone

**DOI:** 10.3390/s25206452

**Published:** 2025-10-18

**Authors:** Bong-Gyu Park, Miso Kim, Jong-Sung Lee, Kwan-Dong Park

**Affiliations:** 1 Department of Geoinformatic Engineering, Inha University, 100 Inha-ro, Incheon 22212, Republic of Korea; geek1206@inha.edu (B.-G.P.); mskim@ppsol.com (M.K.); jslee99@inha.edu (J.-S.L.); 2 PP-Solution Inc., 606 Seobusaet-gil #B-2311, Seoul 08504, Republic of Korea

**Keywords:** smartphone, GNSS, environmental context, C/N_0_, PDOP, ECI

## Abstract

With location-based services becoming more common, smartphone global navigation satellite systems (GNSS) have begun to play a significant role in daily life. Providing reliable location information to smartphone users requires considering localization uncertainty, which varies with the surrounding environment. In this study, we developed an environmental context indicator (ECI) to provide interpretable, continuous information on GNSS observation quality using carrier-to-noise density ratio (C/N_0_), the number of visible satellites, and positional dilution of precision (PDOP). The ECI was developed using a Samsung Galaxy S21+ and satellite signals from global positioning system (GPS) L1/L5, Galileo E1/E5, and BeiDou B1, consisting of three components: a real-valued indicator ranging from 0 to 6, an integer-valued indicator ranging from 1 to 5, and a probability density ratio representing the reliability of the integer-valued indicator. In experimental results, the ECI reflected the variations in the observation environment and corresponding quality changes. ECI values were lowest in open areas, increasing when approaching an urban area, and reaching their maximum in indoor environments where signal reception is severely limited. Consequently, ECI was influenced by building density, exhibiting large and frequent changes, particularly in urban areas.

## 1. Introduction

After the release of the Google Android 7 Nougat operating system in August 2016, raw measurements from a global navigation satellite system (GNSS) became available on smartphones [1]. This technical breakthrough allowed GNSS researchers to expand their studies, previously limited to mass-market GNSS receivers, to include smartphones. As location-based services (LBS) have become more common, smartphone users utilize GNSS location information in various applications, such as maps, social media, logistics, and health care. Thus, LBS are not merely map services but represent a key technology that integrates user location information across diverse applications, supporting personalized, context-aware services and real-time decision-making [2].

Smartphone users employ LBS in various contexts that include indoor and outdoor environments. In particular, the use of LBS has increased in urban areas with high pedestrian traffic and numerous facilities. However, in challenging environments such as urban areas, the accuracy of the smartphone GNSS decreases and uncertainty increases. This is caused by the quality of smartphone GNSS measurements and the multipaths resulting from obstructions [3]. Therefore, stochastic weighting models with a carrier-to-noise density ratio (C/N_0_), satellite elevation angle, and measurement residuals are preferred to address this issue and reduce the impact of low-quality observations on positioning [4,5,6,7]. In addition, by enhancing the filtering method, positioning performance can be improved [8].

Unlike the aforementioned methods, some studies classify the observation environment for context-aware positioning. The observation environment was divided into several categories to train the classification models. In general, observation environment was categorized into broad types such as open, urban, indoor, and vegetated. In some cases, they were further divided into over- and under-viaduct and bridges depending on the purpose. Then, environment-dependent features such as the number of visible satellites, C/N_0_, positional dilution of precision (PDOP), residuals of code-pseudorange and carrier-phase, and Doppler data were collected and calculated. After dataset collection, machine learning models such as support vector machine (SVM), random forest, the hidden Markov model (HMM) [9,10,11,12,13,14], convolutional neural network (CNN), long-short term memory (LSTM), and gated recurrent unit (GRU) were trained [15,16,17,18,19,20,21,22].

Previous studies suggest several classification models; however, the use of classification results for positioning is limited. Zhu et al. [16] trained the LSTM on C/N_0_, the number of visible satellites, PDOP, and code-pseudorange residual, further improving the performance by adjusting the weight model according to the observation environment using the ratio of the time series hidden state. Hussain et al. [23] proposed a context-aware navigation approach that classifies environments into three categories—standard, degraded, and highly degraded—based on satellite visibility. Their study was conducted using a Septentrio PolaRx5s receiver, where C/N_0_ was applied for satellite cut-off and PDOP was used as a performance evaluation metric. They improved positioning performance by adjusting the tracking loops according to the classification results. In contrast, this study focuses on smartphone GNSS, in which signal characteristics differ significantly from those of conventional geodetic receivers. Specifically, C/N_0_ exhibits a weak correlation with satellite elevation angles [24,25,26] but a strong correlation with code-pseudorange residuals [27], making it a key indicator for evaluating the quality of observations in smartphone GNSS.

As mentioned earlier, previous studies have shown that both rule- and artificial intelligence (AI)-based classification methods are effective in enhancing positioning performance. However, smartphone GNSS research has focused on classification using AI technology [9,12,13,14]. Although previous studies have made valuable contributions to improving classification accuracy, most existing approaches have been effective mainly under static conditions or in specific environments and therefore fail to represent the continuously changing observation contexts that occur in dynamic situations. Consequently, these methods may not clearly capture the relationship between GNSS positioning performance and variations in the observation environment. To overcome these limitations, this study does not merely focus on classification. Instead, it revisits the raw GNSS data processing level and proposes an interpretable and real-time applicable approach that leverages key quality indicators—C/N_0_, PDOP, and the number of visible satellites—to support context-aware navigation.

Therefore, we devised an environmental context indicator (ECI) to represent and specify the observation environment in various forms, using GNSS raw measurements from Android smartphones. For ECI development, we utilized the global positioning system (GPS) L1/L5, Galileo E1/E5, and BeiDou B1 signals from Samsung Galaxy S21+. This study used C/N_0_, the number of visible satellites, and PDOP without considering Doppler, carrier phase, and code-pseudorange residuals for ECI. Observation residuals were intentionally excluded to ensure that the ECI remained independent of any specific positioning algorithm. This is because both the magnitude of residuals and the error components vary depending on the positioning method (e.g., standalone or relative positioning). Therefore, by excluding residuals, the ECI can be applied more generally across various positioning techniques. In contrast, C/N_0_, PDOP, and the number of visible satellites are less constrained by the choice of positioning algorithm and were therefore selected as the three representative parameters. The ECI also included categorical information classified by an empirical model and probability information regarding the classification result. The objective was to identify the GNSS observation environment of smartphones and provide detailed information. In addition, through validation, we intended to show that variations in observation quality that depend on the continuously changing observation environment can be detected, and interpretable information can be produced.

### Key Contributions

The contributions of this study can be summarized as follows:

- A raw-data-based, interpretable, and simple framework capable of real-time monitoring of both observation environments and smartphone GNSS quality.
- Utilization of key indicators directly associated with positioning performance—including C/N_0_, PDOP, and the number of visible satellites—for straightforward integration with real-time operation and positioning algorithms.

The remainder of this paper is organized as follows. Section 2 describes the equipment used in this study, the observation environment that defines the ECI, as well as the method of calculating the ECI. Section 3 presents the validation results, and Section 4 provides a discussion of the remaining issues concerning ECI. Section 5 provides a summary and concluding remarks.

## 2. Materials and Methods

### 2.1. Equipment

This subsection describes the smartphone model and the logging configuration. The experiments were conducted using a Samsung Galaxy S21+ and a custom-developed logger currently under development. The detailed specifications are summarized in Table 1.

The Samsung Galaxy S21+ used in this study operated on Android 14 and was equipped with an Exynos 2100 chipset, a Mali-G78 MP14 GPU, and 8 GB of RAM. The device can receive signals from multi-GNSS constellations and supports dual-frequency tracking for GPS and Galileo. The raw data were recorded at 1 Hz using a custom logger. During data logging, the duty cycling was disabled.

### 2.2. Definition of Observation Environment

Among the various observation environments, indoor and open outdoor areas are clearly distinguished even if quantitative criteria are not established; however, without accurate rules, defining various outdoor areas is difficult. Therefore, depending on the scope or purpose of the study, the qualitative criteria of the researcher often contribute to the rationale for selecting an observational environment. For example, in urban areas, the observation environment is defined as open outdoors, urban canyon (UC), boulevard, viaduct, or tunnel [15,16,17]. An observation environment in a comprehensive category can be defined, without being limited to a specific structure, as open-area (OA), semi-outdoor, semi-indoor (SI), or deep indoor (DI) [18,19,20,21,22]. Semi-outdoor canyons can be divided into suburban, semi-urban (SU), and urban canyons [10,11,12,13,14,15,16,17,18,19,20,21].

**Figure 1:**
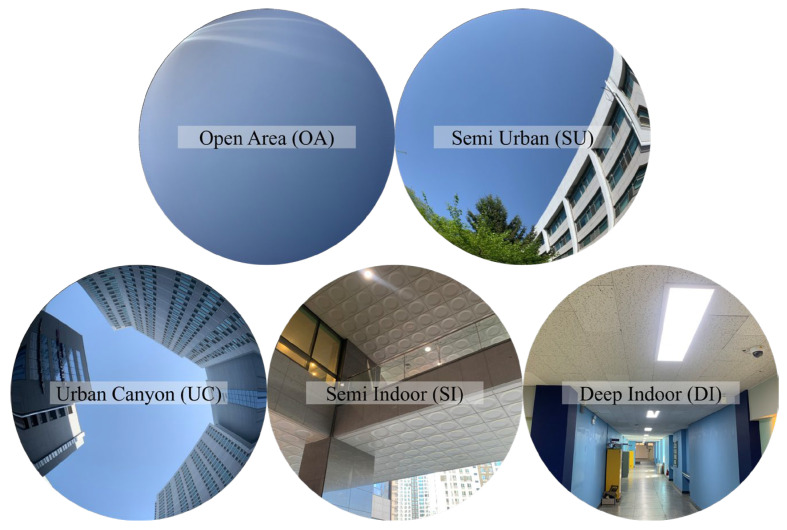
Five representative GNSS observation environments are presented. The Open Area (OA), Semi-Urban (SU), and Urban Canyon (UC) are visualized intuitively by applying a fisheye effect to images captured with a smartphone camera having a 70° field of view. In SU environments, a portion of the sky may be partially obstructed, while a larger portion becomes heavily blocked in UC environments due to surrounding buildings. In contrast, the Semi-Indoor (SI) and Deep Indoor (DI) are displayed without the fisheye effect to allow clearer recognition of the surrounding indoor environment.

In this study, the main purpose was to generate an index that can represent the observation environment and classify it using an empirical model to produce additional information. However, considering the observation environment in excessive detail may increase the uncertainty of the resulting information. Thus, our observation environment comprised the aforementioned OA, SU, UC, SI, and DI, as shown in Figure 1.

Each environment exhibits different characteristics in terms of signal attenuation and satellite visibility, depending on the surrounding buildings or structures. The characteristics of the environments considered in this study are summarized in Table 2.

The OA is hardly affected by the surrounding obstacles and is an environment in which GNSS signal reception is stable. Therefore, severe signal attenuation rarely occurs in this environment. The SU represents an area with a concentration of low-rise buildings, whereas UC refers to an environment in which signal reflection and blockage are more severe because of the presence of high-rise buildings, resulting in increased signal attenuation and reduced satellite visibility. Urban areas are places where the population density is high, and cultural facilities and residential complexes are concentrated. The quality of the signal varies depending on the arrangement and height of the buildings. Thus, in this study, we divided the urban area into SU and UC, based on building height and obstacle. SI refers to a place where a ceiling exists and signal acquisition is possible only through certain spaces. The DI covers the area a few meters from the entrance, and except for the entrance, signal acquisition is almost impossible because there are no windows. Obtaining a GNSS signal and performing positioning are difficult indoors. However, some buildings are made of glass rather than walls; therefore, a signal can be received through the sides even in places where the lower floors are open. Therefore, we divided the indoor environment into SI and DI.

In this study, integer-valued indicators were assigned to each previously defined observational environment for data processing and interpretation, with OS, SU, UC, SI, and DI corresponding to 1, 2, 3, 4, and 5, respectively.

### 2.3. Process of ECI Calculation

**Figure 2:**
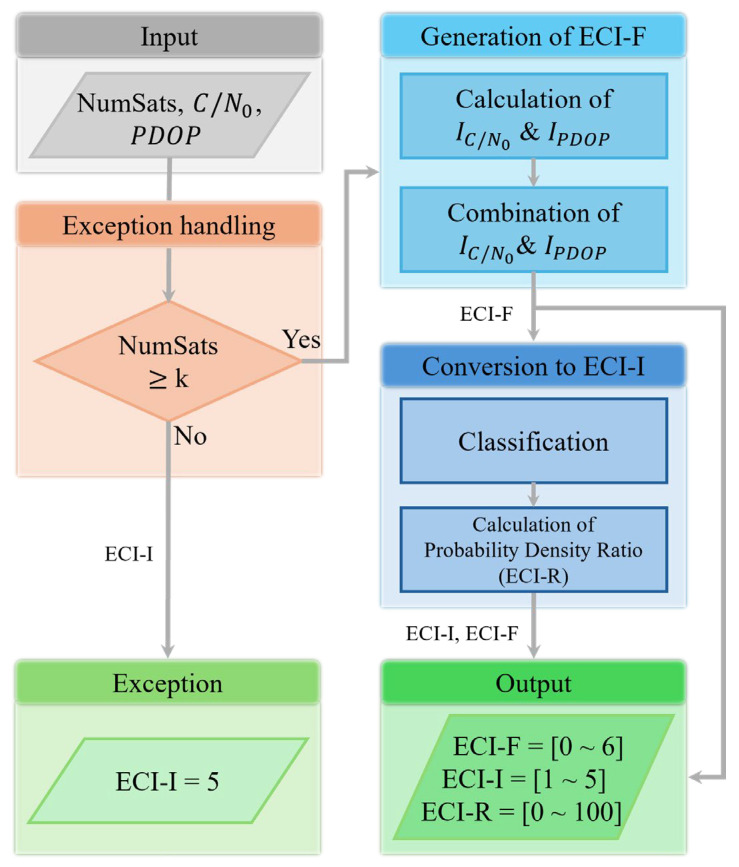
Flowchart of ECI calculation. ECI is generated using the number of satellites, C/N_0_, and PDOP. The process includes exception handling, generation of real-valued ECI (ECI-F), conversion to integer-valued ECI (ECI-I) and derivation of the probability density ratio (ECI-R).

In this section, we describe the overall process of calculating the ECI, detailing specific methods for calculating real- and integer-valued ECI in Section 3.1 and Section 3.2, respectively. The ECI calculation process is illustrated in Figure 2.

The left-hand side of the flowchart shows the exception handling process after receiving the number of satellites, C/N_0_, and PDOP. Exception handling determines whether the number of visible satellites satisfies the minimum number *k* required to perform positioning. Here, k depends on the positioning algorithm to which ECI is applied. For example, if standard point positioning is performed with a single GNSS constellation, k = 4, because three-dimensional (3D) coordinates and receiver clock errors must be estimated. If the number of satellites at a given instant is less than the minimum number of satellites required for positioning, the integer-valued ECI (ECI-I) is determined as 5. If more than *k* satellites are visible, the ECI calculation is performed as shown on the right-hand side of the flowchart, applying pre-processing to C/N_0_ and PDOP, to create real-valued indicators ($I_{C/N_{0}}$, $I_{PDOP}$) within the range of 0–6 by combining $I_{C/N_{0}}$ and $I_{PDOP}$ for real-valued ECI (ECI-F). Subsequently, the real-valued ECI is converted into an integer value ranging from 1 to 5, using an empirical classification model, and the probability density ratio (ECI-R) is calculated to indicate the reliability of ECI-I. Each stage of ECI calculation and its outputs are described in the following subsections.

### 2.4. Generation of ECI-F

**Figure 3:**
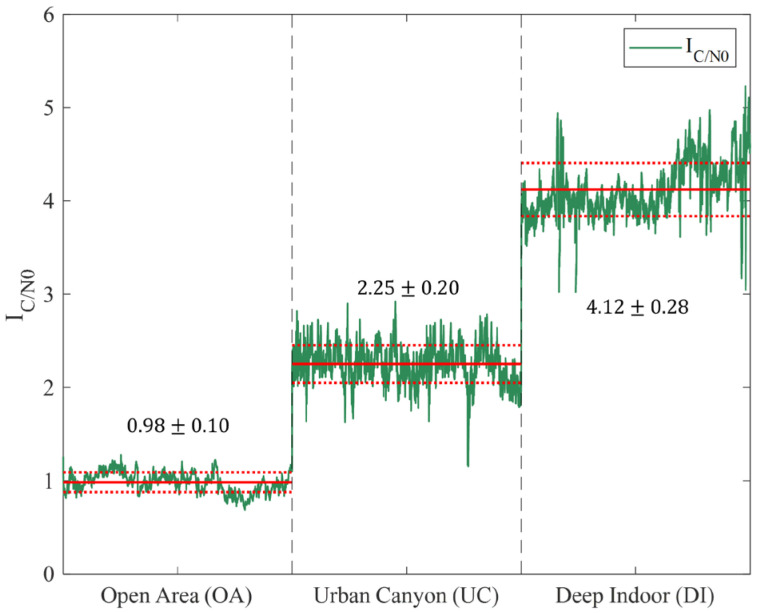
$I_{C/N_{0}}$ in different environments. The average and standard deviation of $I_{C/N_{0}}$ are shown for Open Area (OA), Urban Canyon (UC), and Deep Indoor (DI). Approximately 470,000 measurements were collected in OA, and 300,000 each in UC and DI. From each dataset, 5000 epochs were arbitrarily selected for analysis.

In this subsection, we explain the methods for calculating C/N_0_, PDOP, and ECI-F. The C/N_0_ exhibits different distributions according to the signal-reception conditions. In an outdoor environment, the frequency of C/N_0_ corresponding to the range 30–40 dB-Hz is higher; however, in an indoor environment, the value 20 dB-Hz or less is more frequent, and in urban environments, the frequency is evenly distributed over 20–40 dB-Hz [12]. However, to convert C/N_0_ into a real-valued index, a specific range and criteria are required. Therefore, we collected approximately 470,000 measurements in OS environments such as building rooftops and open fields to establish the criterion for preprocessing. Data were acquired during daytime and nighttime to select various satellite geometries, and average and standard deviation of the C/N_0_ were calculated to establish a reference criterion. The preprocessing formula is as follows:

(1) $$I_{C/N_{0}}=\frac{1}{N}\sum_{i\;=\;1}^{N}\left|\left(C/N_{0}^{i}\left(t\right)-\mu_{ref}\right)/\sigma_{ref}\right|$$

where $\mu_{ref}$ and $\sigma_{ref}$ are the mean and standard deviation of C/N_0_ collected in the OS environment, respectively, and N is the number of satellites observed at a specific epoch t; $C/N_{0}^{i}(t)$ is the C/N_0_ value of the i-th satellite observed at a specific epoch t, and $I_{C/N_{0}}$ is calculated by taking the average of the Z-scores of the satellites observed at each epoch. By calculating the Z-score as the reference statistic of the OS, we can determine how far the C/N_0_ at a specific time t is from the average C/N_0_ of the open area. As an example, we present the $I_{C/N_{0}}$ for OS, UC, and DI in Figure 3.

The green line is the $I_{C/N_{0}}$ calculated for 5000 s in each environment; the red solid line represents the average of $I_{C/N_{0}}$; and the red dotted line represents the standard deviation of $I_{C/N_{0}}$. For OA, the average of $I_{C/N_{0}}$ is 0.98, with a standard deviation of 0.10. For UC, the average is 2.25 with a standard deviation of 0.20, and for DI, the average is 4.12 with a standard deviation of 0.28. In UC and DI, the average of $I_{C/N_{0}}$ increased by 1.27 and 3.14, respectively, compared with that of OA, and the standard deviation increased by a factor of 2 and 2.8, respectively. This implies that $I_{C/N_{0}}$ and its standard deviation increase in environments such as UC and DI, in which the quality of GNSS observation can be greatly reduced.

Dilution of precision is an indicator of the quality of satellite geometry. The PDOP represents the three-dimensional arrangement of satellites, and a smaller PDOP indicates better satellite geometry. In addition, PDOP is inversely correlated with the number of visible satellites and increases significantly as the number of satellites rapidly decreases [34]. Therefore, a natural logarithm scale was applied as follows:

(2) $$I_{PDOP}=ln(PDOP)$$

**Figure 4:**
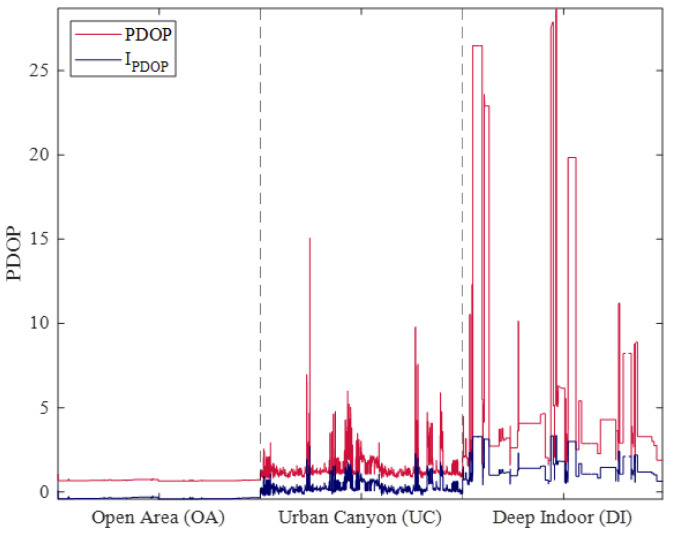
Comparison of PDOP and $I_{PDOP}$ in different environments. The values of PDOP and I_PDOP are presented for Open Area (OA), Urban Canyon (UC), and Deep Indoor (DI). The dataset used for each environment is the same as that used in Figure 3.

To compare the impact of applying the natural logarithm scale, the PDOP and $ln\left(PDOP\right)$ values calculated for the OA, UC, and DI environments are shown in Figure 4.

The red and blue solid lines indicate PDOP and $I_{PDOP}$, respectively. In OA, PDOP slightly varies over time, with magnitude close to 0, but in UC, PDOP changes frequently, with a variation of up to 15. In addition, the variation in PDOP is larger in DI than in UC, and values exceeding 25 are often observed. In contrast, when using a natural logarithm scale, $I_{PDOP}$ shows similar fluctuation patterns but with a smaller magnitude. In UC, the $I_{PDOP}$ varies by up to 2.5 and in DI by up to 4. Figure 3 and Figure 4 suggest that $I_{PDOP}$ and $I_{C/N_{0}}$ increase under harsh observation environments, and their maximums are similar for DI.

**Figure 5:**
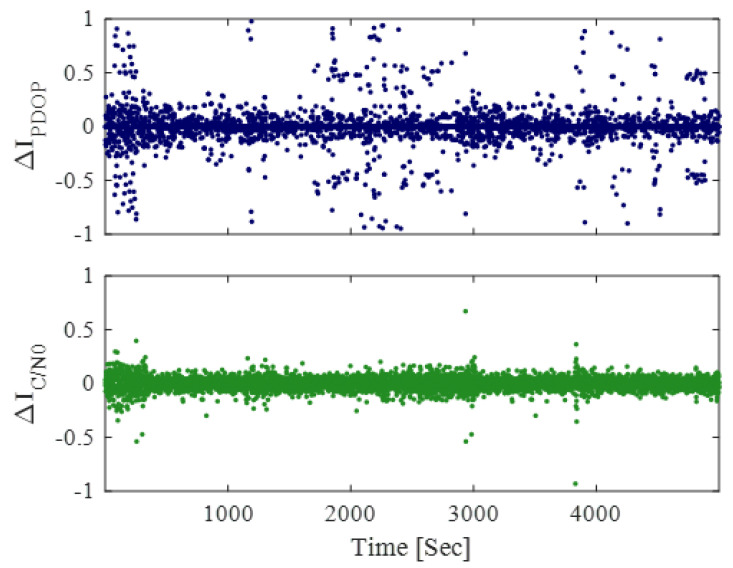
Temporal variations of $I_{C/N_{0}}$ and $I_{PDOP}$ in the Urban Canyon environment. The blue dots represent the variation of $I_{PDOP}\;$(${∆I}_{PDOP}$), and the green solid line represents the variation of $I_{C/N_{0}}$ (${∆I}_{C/N_{0}}$). The data used for calculating these variations are from the same Urban Canyon data shown in Figure 3 and Figure 4.

$I_{C/N_{0}}$ and $I_{PDOP}$ represent the variation in average signal strength and the quality of satellite geometry, respectively. By comparing their temporal variations, it can be observed that combining $I_{C/N_{0}}$ and $I_{PDOP}$ allows the signal strength indicator to also reflect changes in satellite geometry. As shown in Figure 5, $I_{C/N_{0}}$ exhibits several abrupt changes around 3000 s and between 3500 and 4000 s. But it remains relatively stable overall. In contrast, $I_{PDOP}$ shows larger and more frequent fluctuations. This indicates that while $I_{C/N_{0}}$ effectively reflects the variations in average signal strength, it cannot capture changes in satellite geometry. In other words, when the satellite configuration changes significantly due to surrounding buildings or structures, $I_{PDOP}$ responds sensitively, whereas $I_{C/N_{0}}$ alone cannot capture these effects.

Therefore, combining the two indicators enables simultaneous representation of both signal quality and satellite geometry, allowing more robust and accurate detection of environmental changes compared with using a single indicator. Similar approaches have also been reported in previous studies. For instance, Li et al. [35] demonstrated that integrating PDOP into conventional weighting models can effectively reduce positioning errors. The results of this study clearly indicate that the two indicators provide complementary rather than redundant information. To incorporate this characteristic into the ECI-F, a weighted combination method considering the variations in both indicators was adopted.

The weight was calculated based on the amount of change in $I_{C/N_{0}}$ and $I_{PDOP}$, assigning more weight to an index that is more sensitive to changes in the observation environment. The weights of $I_{PDOP}$ ($W_{PDOP}$) and $I_{C/N_{0}}$ ($W_{C/N_{0}}$) were calculated as follows:

(3) $$W_{PDOP}=\frac{1}{1+e^{-\left(\left|∆I_{PDOP}\right|-\left|∆I_{C/N_{0}}\right|\right)}},\;\;W_{C/N_{0}}=1-W_{PDOP}$$

**Figure 6:**
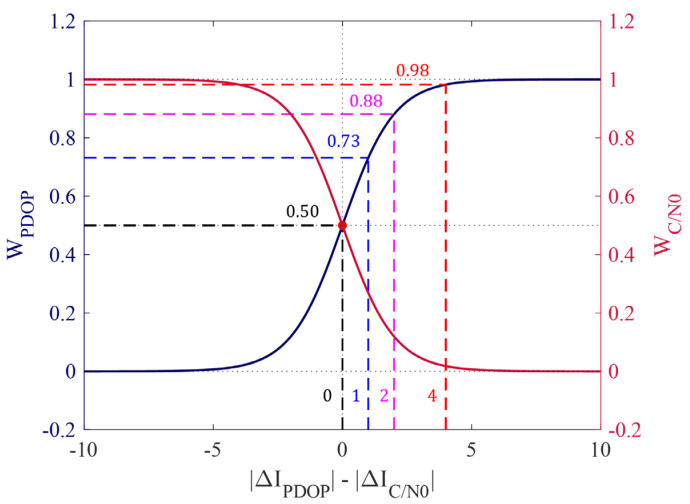
Weighting model for combining $I_{C/N_{0}}$ and $I_{PDOP}$. The model is defined according to the difference between $\left|∆I_{PDOP}\right|$ and $\left|∆I_{C/N_{0}}\right|$. Each dashed line represents $W_{PDOP}$ at difference of 0, 1, 2, and 4.

First, W_PDOP is calculated by using the difference between the changes in the two indicators ($\left|∆I_{PDOP}\right|-\left|∆I_{C/N_{0}}\right|$) as input to the sigmoid function, and $W_{C/N_{0}}$ is calculated as in Equation (3), such that the sum of the weights is 1. To demonstrate the characteristics of the weights, the weight model is shown in Figure 6, along with examples.

When $\left|∆I_{PDOP}\right|-\left|∆I_{C/N_{0}}\right|$ is positive, $W_{PDOP}$ increases, and when $\left|∆I_{PDOP}\right|-\left|∆I_{C/N_{0}}\right|$ is negative, $W_{C/N_{0}}$ increases. When $\left|∆I_{PDOP}\right|-\left|∆I_{C/N_{0}}\right|$ is 0, the two indicators have equal weights of 0.50, as indicated by the black dashed line. When $\left|∆I_{PDOP}\right|-\left|∆I_{C/N_{0}}\right|$ is 1, $W_{PDOP}$ is 0.73, as in the blue dashed line, and $W_{C/N_{0}}$ is 0.27, according to Equation (3). Then, if $\left|∆I_{PDOP}\right|-\left|∆I_{C/N_{0}}\right|$ is 2, $W_{PDOP}$ becomes 0.88, and $W_{C/N_{0}}$ becomes 0.12, according to Equation (3). The sigmoid function converges to 0 as the value along the horizontal axis decreases and converges to 1 as the value along the horizontal axis increases. In Figure 6, even when the $\left|∆I_{PDOP}\right|-\left|∆I_{C/N_{0}}\right|$ is 4, the weight is not 1 but 0.98, which is close to 1. Therefore, even if extreme changes occur in only some indicators, the characteristics of the other indicators still play a role. ECI-F is finally calculated based on this weight model, as expressed in Equation (4)

(4) $$ECI-F=\alpha \left(W_{PDOP}I_{PDOP}+W_{C/N_{0}}I_{C/N_{0}}\right)$$

The ECI-F is calculated by combining the two indicators using the weights as determined by Equation (3). In this case, the scale factor α is used to prevent the scale of the ECI-F from being significantly reduced or increased. The $I_{PDOP}$ can vary greatly depending on the satellite configuration; therefore, it is difficult for the scale to perfectly match $I_{C/N_{0}}$. If $I_{PDOP}$ is smaller than $I_{C/N_{0}}$, the range of ECI-F becomes narrower. Otherwise, if $I_{PDOP}$ is larger than that of $I_{C/N_{0}}$, the range of ECI-F becomes wider. We set α to 2 and the minimum and maximum values to 0 and 6, respectively, to simplify the interpretation of ECI-F.

### 2.5. Conversion to ECI-I

**Figure 7:**
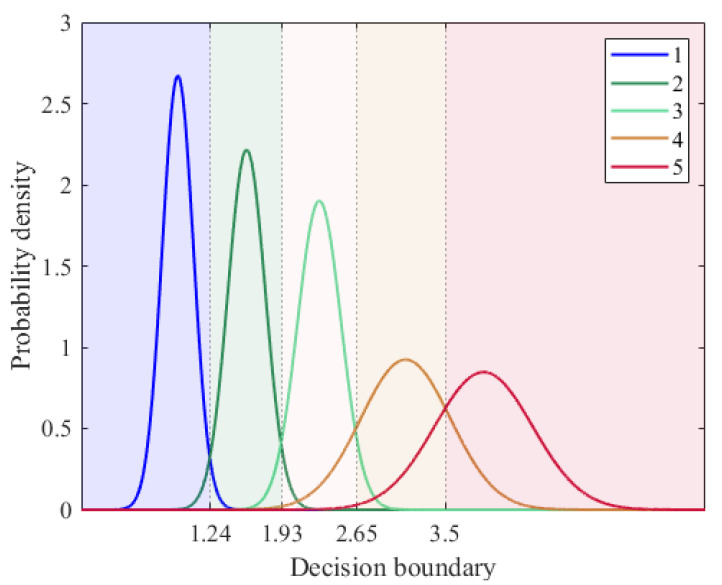
Empirical classification model for conversion to ECI-I. The probability density functions for ECI-I (1–5) are shown with their corresponding decision boundaries, which determine the conversion from ECI-F to ECI-I.

In this subsection, we describe the conversion of ECI-F to ECI-I. Conversion refers to classifying which predefined observation environments correspond to ECI-F. In this study, we constructed an empirical model based on the data collected in advance. According to Equation (1), $I_{C/N_{0}}$ is the average of the Z-scores calculated using the average C/N_0_ and its standard deviation in OA. Notably, $I_{PDOP}$ fluctuated significantly depending on the observation conditions, making it difficult to assume a specific distribution and the corresponding ECI-F for the five observation environments. Therefore, the empirical distribution was calculated under the assumption that $I_{C/N_{0}}$ follows a normal distribution. The normal probability densities corresponding to $I_{C/N_{0}}$ for each observation environment are shown in Figure 7.

During classification, the points with the same probability density were set as decision boundaries. We used ECI-F as the input to the classification model so that $I_{PDOP}$ and $I_{C/N_{0}}$ can affect the classification result. For example, when ECI-F is 2.5, the probability density is higher for indicator 3 than for indicator 4; therefore, ECI-F was assigned to indicator 3, and ECI-I was set to 3. Furthermore, if $I_{C/N_{0}}$ is located at the intersection of the two distributions, the distinction between the two observation environments is unclear. In this case, the effect is an increase in the index according to Equation (3), when the change in $I_{PDOP}$ or $I_{C/N_{0}}$ increases rapidly compared to the previous epoch. When the changes in the two indicators decreased sharply, the lower indicator was selected.

### 2.6. Calculation of ECI-R

In this subsection, we explain the calculation of ECI-R. The ECI-I represents a general category for the observation environment and does not indicate a specific difference in quality within the same category. As a larger ECI-F corresponds to a larger ECI-I, the difference in the quality of a specific ECI-I can be determined using the ECI-F. However, as shown in Figure 7, interpreting the differences near the intersections of adjacent distributions is difficult. At the intersection, even if ECI-I is assigned a specific value, the high probability density of the other indicators makes it difficult to interpret ECI-I. Therefore, ECI-R was calculated according to Equation (5), to indicate the extent to which the other indicators contribute to a specific ECI-I.

(5) $$ECI-R\left(x\right)=\frac{f_{n}\left(x\right)}{f_{1}\left(x\right)+f_{2}\left(x\right)+f_{3}\left(x\right)+f_{4}\left(x\right)+f_{5}\left(x\right)}\times 100$$

**Figure 8:**
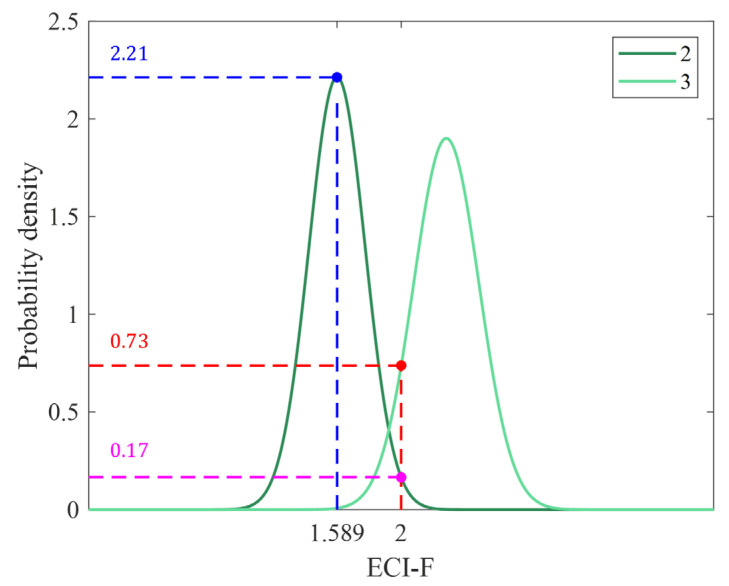
Example of calculating ECI-R when ECI-I is 2 or 3. The probability density functions for ECI-I values of 2 and 3 are shown, and each dash line represents the probability density at the corresponding ECI-F value.

The ECI-R is the percentage of the sum of the probability densities of indicator n=1,...,5 and those of the indicators corresponding to a specific ECI-F, and $f_{n}(x)$ is the probability density function of each indicator n. At each time point, the ECI-R is not a single value; it consists of five density ratios, and their sum equals 100. For a detailed explanation, we provide an example of calculating the ECI-R for indicators 2 and 3 in Figure 8. For convenience, indicators 1, 4, and 5 are not considered in the example.

When the ECI-F is 1.589, the probability density of ECI-I = 2 is 2.21, as indicated by the blue dashed line. By contrast, the probability density of ECI-I = 3 is close to 0. Therefore, the ECI-R of indicator 2 is close to 100. This means that the possibility of ECI-I = 2 is the highest when the ECI-F is 1.589. If ECI-F is 2, the probability density of ECI-I = 3 is 0.73, as indicated by the red dashed line, and that of ECI-I = 2 is 0.17, as indicated by the pink dashed line. In this case, the ECI-R of indicators 3 and 2 are 73% and 17%, respectively, and the ECI-I is 3, with 17% remaining for ECI-I = 2. Therefore, the resulting ECI-R value quantifies the uncertainty of ECI-I at the intersection of adjacent distributions, indicating the reliability of the ECI-I and a detailed quality of the observation environment within the same ECI-I.

## 3. Results

### 3.1. Summary of Test Scenario

This subsection summarizes the three experimental scenarios conducted to validate the ECI. Each experiment was designed to illustrate the characteristics of the ECI under different environmental conditions. The information related to the three experimental scenarios is summarized in Table 3.

In the first experiment, indoor–outdoor transition, a rapid change in satellite visibility occurs. In this scenario, data were first collected in a static state, followed by walking to perform state transitions between indoor and outdoor environments. The walking speed was approximately 0.50 m/s, and a total of 480 s of data were collected.

In the second experiment, conducted in urban and semi-indoor environments, satellite visibility was generally better than in the previous scenario but still experienced significant signal attenuation compared to the open area. In this experiment, static data were collected in an urban environment, followed by walking data in a semi-indoor environment, and this process was repeated. The walking speed was approximately 0.65 m/s, and a total of 504 s of data were collected.

In the final experiment, the test started in an open area and moved into an urban area, resulting in gradual signal attenuation. No static data were collected in this experiment, and only walking data were recorded. The walking speed was approximately 1.30 m/s, and the total duration was 525 s.

The detailed procedures and results of each experiment are described in the following subsections. Section 3.2 presents the indoor–outdoor transition, Section 3.3 describes the urban and semi-indoor walking experiment, and finally, Section 3.4 reports the results of the open-area and urban walking experiment.

### 3.2. Indoor-Outdoor Transition Test

**Figure 9:**
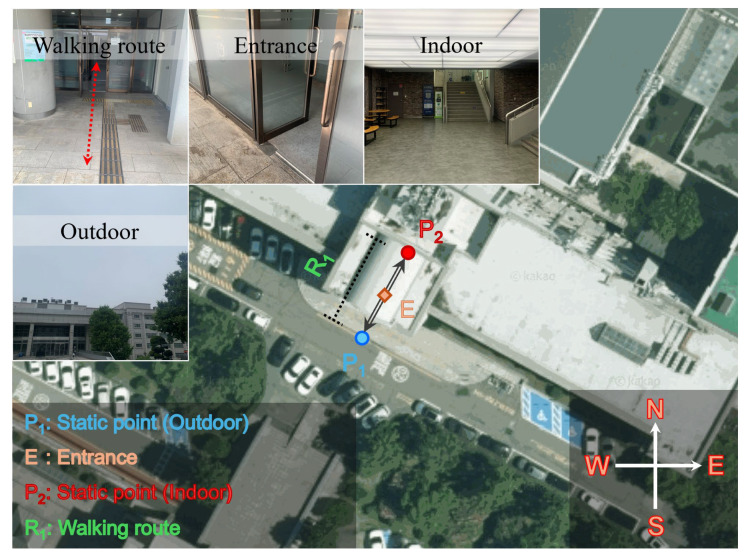
Test environment of indoor-outdoor transition. The test site includes outdoor, entrance, and indoor areas, with photographs illustrating each environment. The experiment consists of an outdoor static point (P_1_), and indoor static (P_2_), an entrance point (E), and a walking route (R_1_). Imagery: ©Kakao Corp. (accessed on 16 June 2025).

This subsection explains the indoor-outdoor transition characteristics of the ECI by presenting experiments involving repeated walking. The experiment was conducted on 16 June 2025, at the southern entrance of the College of Engineering of Inha University. First, the smartphone was held at chest level, and 60 s of data were collected in outdoor areas (P_1_, blue circle), as shown in Figure 9. Subsequently, the subject passed through the building entrance (E, orange rectangle) and entered the indoor areas (P_2_, red circle). In the following step, 60 s of stationary data were collected again at P_2_, and the participant moved back to P_1_ through the same entrance. While collecting kinematic data, the participant walked at approximately 0.50 m/s. This procedure was repeated twice. During the third repetition, data was collected at P_1_. Subsequently, the participant entered P_2_ to collect 60 s of data before completing the experiment. The resulting walking sequence was P_1_ → E → P_2_ → E → P_1_ → E → P_2_ → E → P_1_ → E → P_2_. This yielded three stationary datasets at P_1_ and P_2_, and 120 s of five kinematic datasets during transitions between indoor and outdoor environments through entrance E. The sampling interval of the data was 1 s, and the overall duration of this test was 480 s. All time tags were recorded at the start of walking, passage through the entrance, and end of walking.

**Figure 10:**
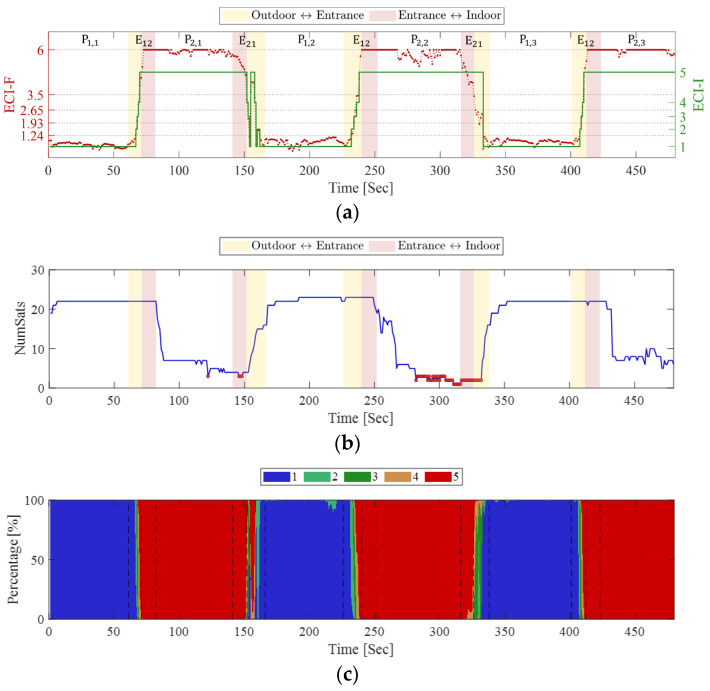
(**a**) ECI-F, ECI-I, (**b**) number of satellites, and (**c**) ECI-R during indoor-outdoor transitions. The path from P_1_ to the entrance or from the entrance to P_1_ is shown as a yellow patch. The path from the entrance to P_2_ or from P_2_ to the entrance, is shown as a red patch.

Here a brief description is provided for each location and path, along with the notation used. First, as shown in the left and top photos of Figure 9, P_1_ is a point approximately 5 m from the entrance. Its upper side is open, allowing for stable collection of satellite signals. This location provides a largely unobstructed sky view, resembling an OA environment. In the middle of the walking route leading to the entrance, signals are likely to be partially blocked or reflected because of an overhang rather than being received directly. Upon entering P_2_, which is located approximately 5 m from the entrance, all parts of the area are enclosed by ceilings and walls, except for the entrance. This weakens the signal strength, and the number of visible satellites is significantly reduced. To distinguish each data acquisition point and path in Figure 10, the three stationary datasets collected at P_1_ are labeled P_1,1_, P_1,2_, and P_1,3_, with the second digit of the subscript indicating the sequence. The path from P_1_ to P_2_ is defined as E_12_. The three static datasets collected at P_2_ are labeled P_2,1_, P_2,2_, and P_2,3_, and the path from P_2_ to P_1_ is denoted as E_21_.

During the experiment, smartphone signals were collected at each point along each path. Based on these data, the calculated ECI-F and ECI-I values, the number of satellites, and ECI-R are shown in Figure 10. To determine whether the walking route is outdoor or indoor relative to the entrance/exit, the path from P_1_ to the entrance or from the entrance to P_1_ is shown as a yellow patch. The path from the entrance to P_2_ or from P_2_ to the entrance, is shown as a red patch. First, examining Figure 10a, during the first 60 s at P_1_, ECI-F remains low at approximately 1, and consequently, ECI-I is also classified as 1. Subsequently, while moving to P_2_ via E_12_, ECI-F reaches approximately 6 within a few seconds whereas ECI-I remains at 5. Next, while moving to P_1_ via E21, ECI-F decreases to below 1.24 within a few seconds. This pattern is consistently observed across all subsequent repetitions.

The number of visible satellites in Figure 10b shows a trend opposite to that of the changes in ECI-F and ECI-I. At P_1_, approximately 20 or more satellites are observed; however, the number decreases sharply when passing through the entrance, reaching less than 10 at P_2_. Thus, the abrupt change in the number of visible satellites during the transition from P_1_ to P_2_ is confirmed to be related to the fluctuations in ECI-F and ECI-I. Furthermore, the areas marked with red dots indicate points where fewer than four satellites are visible, triggering an exception-handling process. This process occurs intensively from the middle of P_2,2_ to the subsequent E_21_ and occurs partially in P_2,1_ and E_21_ following P_2,1_. According to the exception processing rules, ECI-I was determined to be 5, regardless of the ECI-F value.

Finally, in examining the ECI-R in Figure 10c, ECI-I = 1 appears at a rate close to 100% for most of P_1,1_. Subsequently, in the path passing through the entrance, the ratios of ECI-I = 2 or 3 coexist for a short period. Upon arrival at P_2,1_, ECI-I = 5 appears at a rate close to 100%; thereafter, the ratios of ECI-I = 1–4 are mixed in E21. Similar changes persist in the subsequent repetitions. The fluctuation in ECI-R during the indoor-outdoor transition indicates instability in GNSS observations between transitions. Furthermore, the fact that ECI-R for both ECI-I = 1 and ECI-I = 5 is mostly 100% at P_1_ and P_2_ indicates that the ECI clearly distinguishes the environmental differences at P_1_ and P_2_.

Table 4 shows the mean and standard deviation of ECI-F, mode of ECI-I, and mean of ECI-R at each point and path. These data also confirm that ECI clearly distinguishes between indoor and outdoor environments. First, examining the mean of ECI-F, it is 0.97 or lower in all three repetitions at P_1_, and is mostly 6.00 in the three repetitions at P_2_. The standard deviation is below 0.19 at P_1_ and nearly zero at P_2_. That is, at points P_1_ and P_2_, the difference in ECI-F is greater than 5, and the variance is small, making an incorrect determination unlikely. Correspondingly, the mode of ECI-I is 1 at P_1_ and 5 at P_2_.

Regarding ECI-R, ECI-I = 1 accounts for over 93.30% at P_1_, whereas ECI-I = 5 accounts for nearly 100% at P_2_. The ratio of the other indicators, excluding ECI-I = 1 and 5, is at most 6.70%, indicating that ambiguity is nearly absent except during walking. Furthermore, the ratios of ECI-I = 5 at P_1_ and ECI-I at P_2_ are both 0%, indicating that ECI does not cause misclassification after a transition between indoor and outdoor environments.

Additionally, Table 5 confirms that the proposed ECI achieves classification accuracy comparable to that of previous studies in indoor–outdoor transition scenarios. The classification accuracy was evaluated under several conditions: static acquisitions at P_1_ and P_2_ (outdoor and indoor static), walking between P_1_ and the entrance (outdoor → entrance and entrance → outdoor), walking between P_2_ and the entrance (indoor → entrance and entrance → indoor), and the overall scenario. The boundary between indoor and outdoor environments was defined as the entrance. ECI-I values of 1, 2, and 3 were classified as outdoor, while values of 4 and 5 were classified as indoor.

In the outdoor static condition, the classification accuracy reached 100%. During the transition from outdoor to entrance, the accuracy was 72.22%, and from entrance to outdoor, it was 57.14%, indicating relatively lower performance. This reduction is attributed to the presence of an overhang near the entrance, which occasionally causes the ECI to interpret the environment as indoor before entering the indoor space. This implies that in spaces where the boundary between indoor and outdoor environments is clearly distinguishable, even higher accuracy can be achieved. Overall, the ECI achieved 92.15% accuracy in outdoor environments and 100% accuracy under both static and kinematic indoor conditions, resulting in a total accuracy of 96.04% across all scenarios.

Among previous studies that performed indoor–outdoor transition using GNSS data only, Zhu et al. [36] achieved 97.11% accuracy on test data and 99.11% on training data. In their analysis, Gao and Groves [12] reported accuracies of approximately 87–98%, while Wang et al. [9] demonstrated that machine learning algorithms such as SVM and Random Forest could achieve accuracies in the range of 94–98%. Although most of these previous studies employed machine learning–based approaches, the proposed method in this study achieved comparable performance using a non–machine-learning approach.

### 3.3. Static-Kinematic Test in Urban and Semi-Indoor Environment

**Figure 11:**
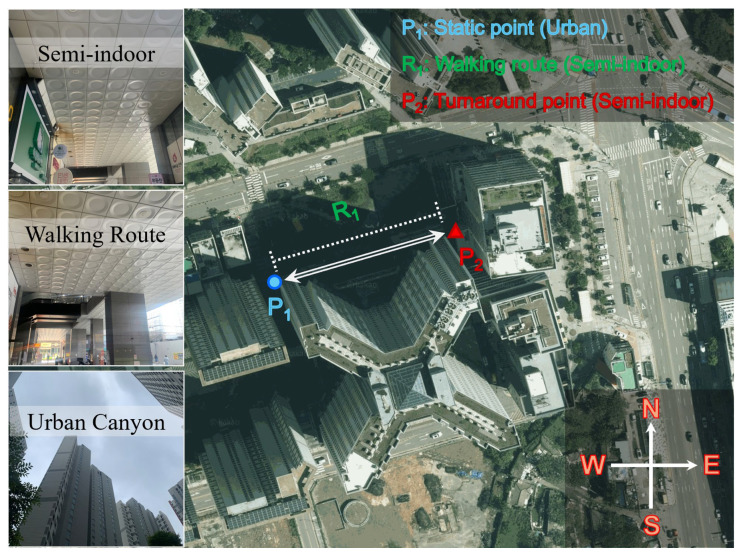
Test environment of urban and semi-indoor walking. The test site includes semi-indoor, walking route, and urban canyon areas, with photographs illustrating each environment. The experiment consists of a static point (P_1_) in the urban area, a turnaround point (P_2_) in the semi-indoor area, and a walking route (R_1_). Imagery: ©Kakao Corp. (accessed on 17 June 2025).

This subsection demonstrates that ECI reflects the changes in the GNSS observation environment and variations in observation quality by presenting an experiment that starts at a point in the UC environment and passes through SI. The experiment was conducted on 17 June 2025, in an apartment complex near Inha University. First, while holding the smartphone at chest level, stationary data were collected for 60 s at an urban location (P_1_, blue circle), as shown in Figure 11. Subsequently, the participant entered a semi-indoor walking route (R_1_) on the ground floor of the building. Next, the participant walked to the turnaround point (P_2_, red triangle), which is approximately 70 m away from P_1_ and immediately returned to P_1_ without collecting additional static data. The return path followed the same route from P_1_ to P_2_. During kinematic data collection, the participant walked at an average speed of approximately 0.65 m/s. Finally, upon returning to P_1_, 60 s of static data were collected again. This procedure was repeated twice. During the third repetition, the experiment was completed at the end of R_1_ while returning from P_2_ to P_1_. The final walking sequence was P_1_ → R_1_ → P_2_ → R_1_ → P_1_ → R_1_ → P_2_ → R_1_ → P_1_ → R_1_ → P_2_ → R_1_. Three sets of static data were collected at P_1_, and three sets of kinematic data were collected at R_1_. In other words, P_1_ is the point at which static data were collected for 60 s each time, kinematic data were collected at R_1_ for approximately 108 s each time, and P_2_ was the turnaround point with no stationary data. The sampling interval was set to 1 s, and the overall duration of this test is 504 s. To facilitate analysis, time tags were used to distinguish the stationary data at P_1_ from the kinematic data at R_1_.

Here, each point and path are briefly described, summarizing the notation used to represent them. As shown in the lower-left photo of Figure 11, P_1_ is surrounded by high-rise buildings, allowing stable observations only near the zenith direction. The signals received are primarily those reflected from the building sides, resulting in a GNSS observation environment of lower quality than in open areas. Next, upon entering R_1_, as shown in the upper and middle sections of the left photo, the path is covered by a canopy-like structure overhead, and the south side is blocked by a wall. This can increase the acquisition of reflected signals, potentially reducing the signal strength. However, the northern side of R_1_ has open lateral space; therefore, a sharp decrease in the number of visible satellites is not expected. P_2_, the turnaround point for R_1_, is the same semi-indoor environment as R_1_, as shown in the upper-left photo. However, P_2_ has a structure that partially blocks the northern direction, potentially causing more severe signal degradation. When returning from P_2_ to P_1_, the same path was followed; therefore, the environmental characteristics remained identical. To distinguish each segment, the three stationary datasets collected at P_1_ were labeled P_1,1_, P_1,2_, and P_1,3_, with the second digit of the subscript indicating the sequence. The three round trips conducted at R_1_ are denoted as R_1,1_, R_1,2_, and R_1,3_.

**Figure 12:**
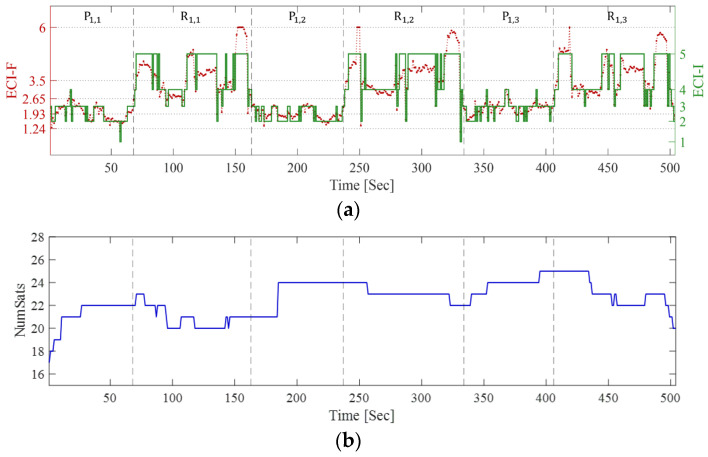

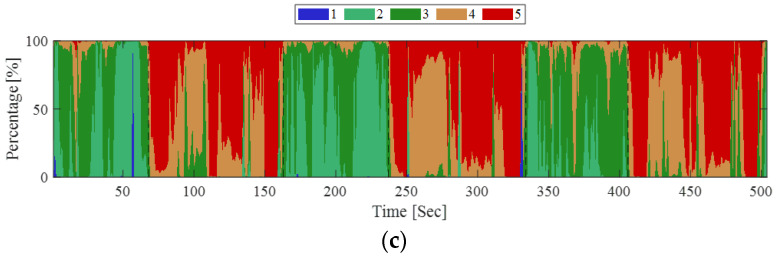
(**a**) ECI-F, ECI-I, (**b**) number of satellites, and (**c**) ECI-R during urban and semi-indoor walking. Each dashed line represents the boundary between static points (P_1,1_, P_1,2_, and P_1,3_) and walking routes (R_1,1_, R_1,2_, and R_1,3_).

The ECI-F and ECI-I values calculated at each point along the path, number of satellites, and ECI-R are shown in Figure 12. In the figure, the time tags marking the start and end of walking at R_1_ are indicated by black dashed lines. First, examining ECI-F and ECI-I in Figure 12a, ECI-F is approximately 1.93 at point P_1,1_ and increases to approximately 3.5 while moving to R_1,1_. During the subsequent walk at R_1,1_, ECI-F fluctuates and then returns to approximately 1.93 upon reaching P_1,2_. This trend is similar in the remaining two repetitions. Notably, unlike previous indoor-outdoor transitions, continuous fluctuations occur throughout all the sequences. ECI-F and ECI-I repeatedly increase and decrease, respectively, during the three P_1_ sequences. Notably, during the three walking sequences at R_1_, ECI-F and ECI-I increase sharply because of the canopy, and the fluctuations become even greater in open space and in the presence of obstacles.

The changes in the number of visible satellites are shown in Figure 12b. Although signals from approximately 20 satellites were received, relatively high fluctuations in the satellite numbers were observed only for R_1,1_ and R_1,3_. This indicates that the signals were being continuously received in open space, and some satellites were temporarily not observed. However, these changes alone are insufficient to discern subtle variations in signal quality. Looking at ECI-R in Figure 12c, the ratios for ECI-I = 2 and 3 are high in the three P_1_ sequences, whereas the ratios for ECI-I = 4 and 5 are high in all sequences of R_1_. Notably, although the ECI-R in the three R_1_ sessions showed similar temporal variations, the detailed ratios changed with each repetition. This indicates that even with small fluctuations in the number of visible satellites, variations in the GNSS observation quality can occur at the same location and path over time.

These variations in observation quality were confirmed by examining the mean and standard deviation of ECI-F, mode of ECI-I, and mean of ECI-R, as shown in Table 6. First, the mean value of ECI-F ranges from 1.93 to 2.18 across the three P_1_ sequences, indicating that the overall observation quality was lower than in open area environment. Furthermore, the increase to 3.68–3.76 at R_1_ indicates that the environment degraded as walking commenced. The standard deviation increases to 0.26–0.36 at P_1_ compared to that in the open area environment, and at R_1_, it reaches 0.96–1.02, which is more than three times that of P_1_. This demonstrates that the observation environment fluctuated significantly over time due to the influence of the surrounding structures during walking.

The ECI-R allows the assessment of environmental variability. At P_1,1_, the predominant indicators are ECI-I = 2 (42.54%) and ECI-I = 3 (48.46%), differing by approximately 6%. A similar pattern is observed in P_1,2_ at 53.86% and 42.05%, respectively. In P_1,3_, ECI-I = 3 has a high proportion at 66.14%, with coexisting ECI-I = 2 (20.83%) and ECI-I = 4 (12.25%). This demonstrates that uncertainty exists when multiple indicators coexist irregularly even in a stationary state.

In the R_1_ sequences, the ECI-I values of 4 and 5 dominate. In R_1,1_, they account for 36.21% and 53.73%, respectively, with ECI-I = 5 exceeding 50%. R_1,2_ also shows nearly identical trends, with proportions of 33.99% and 57.77%, respectively, and R_1,3_ of 34.96% and 55.91%. R_1_ exhibited strong indoor-like characteristics, with ECI-I = 5 comprising more than half, and ECI-I = 4 comprising more than 30%. Notably, although ECI-I is classified as 5 in R_1_, which is similar to the indoor sequences in the previous indoor-outdoor transition experiment, the composition ratio of ECI-R differed significantly between the two environments. Specifically, ECI-I = 5 accounts for nearly 100% with almost no uncertainty in the indoor-outdoor transition case; ECI-I = 4 and 5 coexist in this experiment. This demonstrates that changes in the observation environment and quality over time are represented through various indicators rather than a single absolute metric, even under stationary conditions.

### 3.4. Kinematic Test in Open Area and Urban Areas

This subsection demonstrates that the ECI reflects changes in observation quality as signal blockage intensifies by presenting an experiment for the case of moving from an open terrain to an urban area. The experiment was conducted on 17 June 2025, in the sports field of Inha University and a nearby apartment complex. First, while holding a smartphone at chest level, we walked along the sidewalk from the starting point (blue rectangle) of R_1_ (blue line) to the starting point (yellow rectangle) of R_2_ (yellow line), and then continued to walk along R_2_. At the end of R_2_, we moved to the starting point (red square) of R_3_ (red line) in the apartment complex. Walking continued along R_3_ until the end of the experiment (red star). Unlike the previous experiments, this experiment did not collect static data at the start and end of each path; instead, walking was performed continuously. Consequently, the path was R_1_ → R_2_ → R_3_. The length of R_1_, R_2_, and R_3_ are approximately 185 m, 268 m, and 240 m, respectively, and the walking speed was approximately 1.3 m/s. The sampling interval was 1 s, and the overall duration of this test was 525 s. A time tag was recorded each time the starting point of a path was passed to distinguish between the paths.

**Figure 13:**
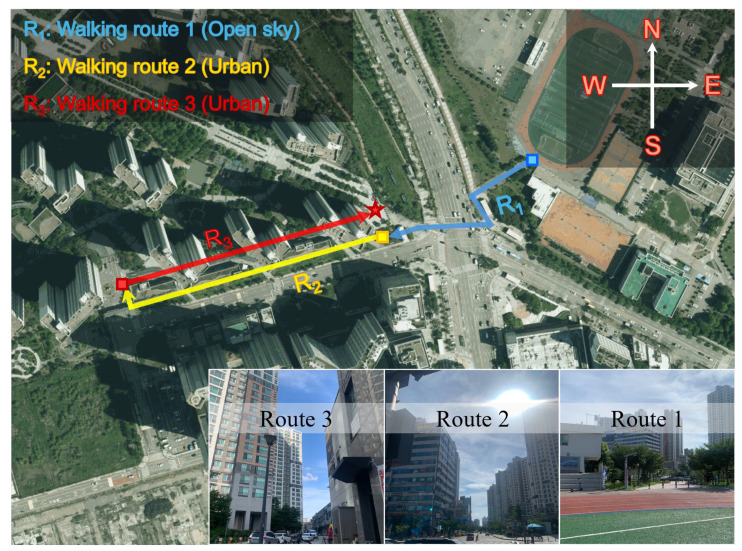
Test environment of open area, urban walking. The test site includes open areas and urban canyon areas, with photographs illustrating each walking route: open area (R_1_), urban (R_2_, R_3_). Imagery: ©Kakao Corp. (accessed on 17 June 2025).

This paragraph provides a brief description of each point and path, along with the notation used to represent them. First, to demonstrate the gradual change in ECI, an entire path was constructed in order of decreasing signal blockage effects caused by buildings. As shown in the lower-right photo of Figure 13, R_1_ has fewer surrounding high-rise buildings, enabling stable signal acquisition. This condition offers favorable visibility for securing multiple satellites and minimal multipath effects, resulting in high-quality GNSS observations. In contrast, R_2_ has high-rise buildings positioned on both sides of the walking path, causing partial signal blockage and reflection in north–south directions. Consequently, the observation quality can deteriorate significantly compared to that of R_1_. While walking on R_2_, the presence of a roadway along the path necessitated the use of a sidewalk adjacent to buildings, potentially causing significant observation degradation because of the obstacles between pedestrians. Near the end of R_2_, an open area facing southwest reduced the influence of buildings and temporarily improved observation quality. Finally, when approaching R_3_, the walking path became narrower than that in R_2_, with a higher proportion of reflected signals, which may further degrade the observation quality. In summary, the impact of signal blockage by buildings intensified progressively from R_1_ to R_3_ across the three paths in this experiment, with ECI fluctuations observed due to blockage while walking. Because the paths were not repeated in this experiment, data from each path are presented as R_1_, R_2_, and R_3_ without the second subscripts to indicate the sequence.

**Figure 14:**
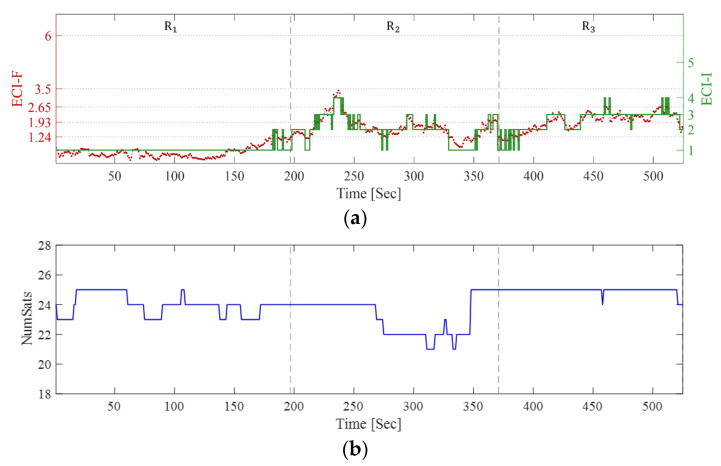

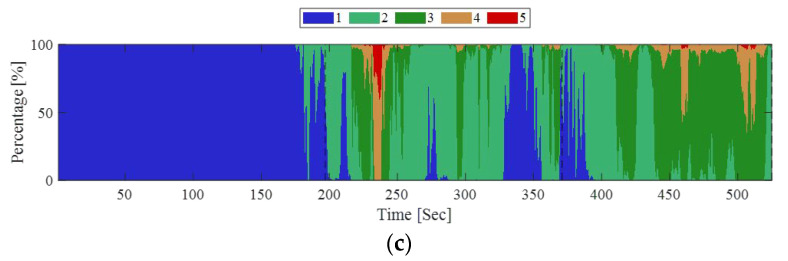
(**a**) ECI-F, ECI-I, (**b**) number of satellites, and (**c**) ECI-R during open area and urban walking. Each dashed line denotes the change in walking route.

During the experiment, the smartphone signals were collected along each path. Figure 14 shows the calculated ECI-F and ECI-I values, number of satellites, and ECI-R based on these signals. In the Figure, the time tag at the points where the walking path changes are indicated by a black dashed line. First, examining ECI-F and ECI-I in Figure 14a, ECI-F remains low at approximately 0.5 in R_1_, and ECI-I is also stably classified as 1. As time progresses and the participant enters the densely built-up area R_2_, the ECI-F exceeds 1.24, and ECI-I is primarily classified as 2 or 3. Subsequently, after the point where ECI-F temporarily increases to approximately 3.5, it fluctuates within the range of 1.24 to 1.93. Moving toward the end of R_2_, the influence of open spaces causes ECI-F to drop below 1.24, and ECI-I to decrease to 1. Finally, upon entering R_3_, ECI-F increases to approximately 1.93, and ECI-I is primarily 3.

The changes in the number of satellites are shown in Figure 14b. Approximately 20 or more satellites were observed on all routes without any abrupt decrease, unlike the indoor transition. However, as shown for ECI-R in Figure 14c, the proportion of ECI-I = 2 increases when the route changes from R_1_ to R_2_ although the number of visible satellites remains the same. Furthermore, while walking in R_2_, ECI-I is mostly 2, whereas ECI-I = 3, 4, and 5 appear only temporarily. In R_3_, the ECI-I = 3 ratio dominates, along with other indicators. This demonstrates that ECI reflects the uncertainty and variability of signal quality inherent to urban environments.

This behavior can also be confirmed through the mean and standard deviation of ECI-F, mode of ECI-I, and mean of ECI-R, as shown in Table 7. The mean value of ECI-F is 0.47 in R_1_, which is lower than that of P_1_ in Table 4, and that of ECI-I is 1. This indicates that R_1_ is closer to an open area, where stable signal reception is possible. However, it gradually increases to 1.63 in R_2_ and 1.93 in R_3_, with ECI-I reaching 2 in R_2_ and 3 in R_3_. This demonstrates that even minor changes in location within an urban environment affect signal quality. Furthermore, the standard deviation is 0.21 in R_1_ but increases to 0.41 in R_2_ and 0.50 in R_3_. This indicates that the variability in GNSS observation quality over time is greater in urban environments than in open terrain.

Regarding ECI-R, the ECI-I = 1 in R_1_ accounts for 99.26%, indicating almost no uncertainty. However, in R_2_, ECI-I = 2 accounts for 56.73% and ECI-I = 1 for 21.42%, with the remaining indicators also showing mixed proportions. In R_3_, ECI-I = 2 decreases to 34.59%, whereas ECI-I = 3 increases to 51.12%, indicating a more challenging observation environment than R_2_. Furthermore, R_3_ exhibits mixed proportions across other indicators, as observed in R_2_. This demonstrates that as the surrounding environment becomes more complex, no single ECI dominates; instead, multiple indicators coexist under uncertain conditions.

## 4. Discussion

A limitation of this study is that the ECI was validated using only the Samsung Galaxy S21+ device. This approach assumed that, while the absolute observation quality may vary across different smartphone models, the degradation pattern of observation quality with respect to the surrounding environment would remain similar. However, recent studies have reported significant differences in C/N_0_ values between smartphone models such as the Pixel 5 and Pixel 9 Pro XL [33], suggesting that device-specific C/N_0_ characteristics may affect the robustness of the ECI. Therefore, further research is required to verify the general applicability of the ECI across different devices and to assess the necessity of device-specific calibration.

Another limitation of this study is that the ECI was trained without distinguishing the C/N_0_ by satellite constellation or frequency band. Instead, at each epoch, the C/N_0_ values of all available satellites were converted to Z-scores and then averaged. This approach was adopted to mitigate the risk of biased outcomes that could arise from the influence of outliers. When the data are divided by constellation or frequency, the reduced number of samples makes the results more sensitive to such effects. However, the characteristics of C/N_0_ can differ significantly across constellations and frequency bands, and such differences may be more significant in smartphones. Therefore, future research should investigate whether separating or combining C/N_0_ by constellation and frequency offers greater benefits for applying the ECI to positioning algorithms.

In this study, five environmental categories—Open Area, Semi-Urban, Urban Canyon, Semi-Indoor, and Deep Indoor—were defined to provide supplementary context for interpreting and applying the ECI-F. The detailed types of labels provide valuable contextual information for developing algorithm calibration and positioning strategies. At the same time, as suggested by Hussain et al. [23], a three-level classification scheme consisting of standard, degraded, and highly degraded conditions is also intuitive, and the proposed ECI-F can be easily simplified and applied to these three categories. Although dividing the environment into five classes may slightly increase the possibility of misclassification near the decision boundaries, this is a common limitation in classification problems and can be mitigated in future studies through the application of AI techniques.

## 5. Conclusions

In this study, we proposed an environmental context indicator (ECI) using C/N_0_ and PDOP from Samsung Galaxy S21+ to quantify the quality of the smartphone GNSS observation environment. The ECI was defined for five environments: open area, semi-urban, urban canyon, semi-indoor, and deep indoor and consisted of three components: a real-valued ECI created by a weighted combination of C/N_0_ and PDOP, an integer-valued ECI generated by an empirical classification model, and a probability density ratio representing the reliability of an integer-valued ECI. Unlike previous studies, this work focused on processing raw data to quantify the observation environment and interpret it as a time series. The indoor-outdoor transition test demonstrated that the ECI effectively distinguishes between indoor and outdoor environments. Moreover, the results show that the ECI reflects continuous changes in GNSS observation quality in harsh environments, such as urban and indoor conditions.

Future studies will focus on refining the ECI to address the limitations and issues discussed in Section 4. Once improved, the ECI can be applied to the weight model, enabling ECI-based adaptive weight adjustment in positioning algorithms. Furthermore, monitoring time-series ECI can support the design of adaptive positioning strategies by detecting abrupt changes in the GNSS observation environment.

## Figures and Tables

**Table 1:** Smartphone and logging configuration.

Category	Specification
Device	Samsung Galaxy S21+ (Samsung Electronics Co., Ltd., Suwon, Republic of Korea)
OS version	Android 14
SoC/CPU	Exynos 2100 (1 × Cortex-X1 ~2.9 GHz + 3 × A78 ~2.8 GHz + 4 × A55 ~2.2 GHz, Samsung Electronics Co., Ltd., Suwon, Republic of Korea)
GPU/RAM	Mali-G78 MP14, 8 GB RAM
GNSS constellations	GPS, Galileo, BeiDou, GLONASS, QZSS
GNSS signals	L1/E1/B1/G1 + L5/E5a
Logging app/rate	Custom-build logger/1 Hz
Duty cycling	Off

**Table 2:** Characteristics of five representative environments. For each of the environments, the table summarizes the description of the observation environment, the typical range of C/N_0_ variation, and the number of visible satellites in multi-GNSS constellations.

Environment	Description	Representative C/N_0_ Range (dB-Hz)	Satellite Visibility (Multi-Constellation)	References
Open area	Outdoor area with sparse obstacles	35–45	18–25	[4,9,28,29]
Semi urban	Densely built-up area with low-rise buildings	20–40	12–20	[30,31]
Urban canyon	Densely built-up area with high-rise buildings	20–35	10–18	[12,31,32]
Semi indoor	Partially covered outdoor spaces/Partially opened indoor space	<32	8–15	[33]
Deep indoor	Indoor area with limited visibility	<25	0–8	[9,12]

**Table 3:** The scenarios, data types, walking speeds, test durations, and characteristics of each experiment are summarized. The sampling interval of the acquired data was 1 s.

Scenario	Characteristics	Data Type	Walking Speed (m/s)	Duration (s)
Indoor-outdoor transition	Abrupt changes in satellite visibility	static, kinematic	≈0.50	480
Urban, semi-indoor walking	severe signal degradation	static, kinematic	≈0.65	504
Open area, urban walking	gradual signal degradation	Kinematic	≈1.30	525

**Table 4:** Test statistics of indoor-outdoor transition. The table presents the mean, standard deviation of ECI-F, mode of ECI-I, along with the average of ECI-R (ECI-I = 1–5), for outdoor static points (P_1,1_, P_1,2_, and P_1,3_) and indoor static points (P_2,1_, P_2,2_, and P_2,3_).

Metrics	P_1,1_	P_1,2_	P_1,3_	P_2,1_	P_2,2_	P_2,3_
Mean of ECI-F	0.78	0.97	0.97	6.00	5.98	6.00
Std. of ECI-F	0.13	0.19	0.11	0.00	0.07	0.00
Mode of ECI-I	1	1	1	5	5	5
Avg. of ECI-I = 1 (%)	99.93	93.30	98.20	0.00	0.00	0.00
Avg. of ECI-I = 2 (%)	0.07	6.70	1.80	0.00	0.00	0.00
Avg. of ECI-I = 3 (%)	0.00	0.00	0.00	0.00	0.00	0.00
Avg. of ECI-I = 4 (%)	0.00	0.00	0.00	0.15	0.13	0.15
Avg. of ECI-I = 5 (%)	0.00	0.00	0.00	99.85	99.87	98.85

**Table 5:** Indoor-outdoor transition accuracy. The entire scenario was divided into detailed segments, and the accuracy for each segment, as well as the overall accuracy, was presented.

Segment	Accuracy (%)		
Outdoor static	100.00%	92.15%	96.04%
Outdoor → Entrance	72.22%		
Entrance → Outdoor	57.14%		
Indoor static	100.00%	100.00%	
Indoor → Entrance	100.00%		
Entrance → Indoor	100.00%		

**Table 6:** Test statistics of urban, semi-indoor walking. The table summarizes the mean, standard deviation of ECI-F, mode of ECI-I, together with the average of ECI-R (ECI-I = 1~5), for static points (P_1,1_, P_1,2_, and P_1,3_) and walking routes (R_1,1_, R_1,2_, and R_1,3_).

Metrics	P_1,1_	P_1,2_	P_1,3_	R_1,1_	R_1,2_	R_1,3_
Mean of ECI-F	1.98	1.93	2.18	3.68	3.75	3.76
Std. of ECI-F	0.36	0.26	0.29	0.97	1.02	0.96
Mode of ECI-I	3	2	3	5	5	5
Avg. of ECI-I = 1 (%)	1.66	0.05	0.00	0.00	1.01	0.00
Avg. of ECI-I = 2 (%)	42.54	53.86	20.83	0.98	2.15	0.01
Avg. of ECI-I = 3 (%)	48.46	42.05	66.14	9.08	5.08	9.12
Avg. of ECI-I = 4 (%)	6.96	3.88	12.25	36.21	33.99	34.96
Avg. of ECI-I = 5 (%)	0.38	0.16	0.78	53.73	57.77	55.91

**Table 7:** Test statistics of open area, urban walking. The table summarizes the mean, standard deviation of ECI-F, mode of ECI-I, as well as the average of ECI-R (ECI-I = 1–5) for the three walking routes (R_1_, R_2_, and R_3_).

Metrics	R_1_	R_2_	R_3_
Mean of ECI-F	0.47	1.63	1.93
Std. of ECI-F	0.21	0.41	0.50
Mode of ECI-I	1	2	3
Avg. of ECI-I = 1 (%)	99.26	21.42	7.30
Avg. of ECI-I = 2 (%)	0.74	56.73	34.59
Avg. of ECI-I = 3 (%)	0.00	16.12	51.13
Avg. of ECI-I = 4 (%)	0.00	4.63	6.72
Avg. of ECI-I = 5 (%)	0.00	1.10	0.26

## Data Availability

The data generated in this study are available from the corresponding author upon reasonable request.

## Abbreviations

The following abbreviations are used in this manuscript:

GNSS	Global Navigation Satellite System
LBS	Location-Based Services
ECI	Environmental Context Indicator
C/N_0_	Carrier-to-Noise Density Ratio
PDOP	Position Dilution of Precision
AI	Artificial Intelligence
GPS	Global Positioning System
HMM	Hidden Markov Model
SVM	Support Vector Machine
CNN	Convolutional Neural Network
LSTM	Long-Short Term Memory
GRU	Gated Recurrent Unit
OA	Open Area
SU	Semi Urban
UC	Urban Canyon
SI	Semi Indoor
DI	Deep Indoor
ECI-F	Real-valued ECI
ECI-I	Integer-valued ECI
ECI-R	Probability Density Ratio
3D	Three-Dimensional
